# Supplementary material for: What to consider before prescribing inhaled medications: a pragmatic approach for evaluating the current inhaler landscape
Source: Ther Adv Respir Dis. 2019 Dec 6;13:1753466619884532. doi: 10.1177/1753466619884532 (PMC6900625; doi:10.1177/1753466619884532)
Supplement: Reviewer_1_v.1 – Supplemental material for What to consider before prescribing inhaled medications: a pragmatic approach for evaluating the current inhaler landscape [file Reviewer_1_v.1.pdf]

Reviewer 1 v.1

Comments to the Author

This is a very comprehensive review on an important clinical topic.

The ms is well written and well referenced.

I agree with all statemnets and analyses.

Three practical points to add:

1. Is there an advantage of once daily dosing versus twice daily?

2. What are the recommendations about breh holding? Is it similar for DPIs and pMDIs?

3. What is the recommende breathing pattern through a VHC?
