# Supplementary material for: What to consider before prescribing inhaled medications: a pragmatic approach for evaluating the current inhaler landscape
Source: Ther Adv Respir Dis. 2019 Dec 6;13:1753466619884532. doi: 10.1177/1753466619884532 (PMC6900625; doi:10.1177/1753466619884532)
Supplement: Reviewer_2_v.1 – Supplemental material for What to consider before prescribing inhaled medications: a pragmatic approach for evaluating the current inhaler landscape [file Reviewer_2_v.1.pdf]

Reviewer 2 v.1

Comments to the Author

This is a well written and comprehensive review of the literature regarding the challenges in using a variety of inhalation devices and considerations for personalizing therapy. The paper is written mainly from a European perspective. Tables 3 and 4 are especially helpful to compare the characteristics of various inhalers and the common errors encountered in clinical practice. Clinicians are likely to benefit from the information in this article.

The authors stress the importance of the effects of environmental conditions on storage and shelf-life of various inhalers. This useful information may be new for many readers.

Inclusion of pictures of various inhalers would enhance the review. Providing links to websites with the correct technique of use would also be helpful.
