# Supplementary material for: What to consider before prescribing inhaled medications: a pragmatic approach for evaluating the current inhaler landscape
Source: Ther Adv Respir Dis. 2019 Dec 6;13:1753466619884532. doi: 10.1177/1753466619884532 (PMC6900625; doi:10.1177/1753466619884532)
Supplement: Reviewer_3_v.1 – Supplemental material for What to consider before prescribing inhaled medications: a pragmatic approach for evaluating the current inhaler landscape [file Reviewer_3_v.1.pdf]

Reviewer 3 v.1
Comments to the Author

General comments

1. It has been a pleasure to review this manuscript. It covers a large body of evidence and hence I would consider it a highly ambitious review. It covers a lot of topics/concept relevant to inhaler use and I commend the authors for undertaking it. However, as a result of its broad scope, I think it does suffer from a lack of direction and hence I think a more strategic and structure approach to its composition and flow is required. I have included some suggestions which I hope will be helpful.
2. At times I feel it is detailed, at times, incomplete.
3. At times it appears that the authors lack focus and stray from what their title suggests this review is about – a pragmatic approach and what to consider when prescribing.
4. Finally, what I feel it is lacking is an evidence-based series of recommendations, which is what is most needed in this field of research and practice. My question is, what will health care providers get out of this with regards to selecting devices? Will device selection now be more systematic? Will it be more individualised? Or will health care professionals simply be more informed? At this stage it is a literature review of a series of topics, often a literature review of secondary sources (e.g Table 1 and its reference to two review manuscripts).
5. There has been explosion of manuscripts on inhaler technique and devices over the last 5-7 years in particular. Further publications really need to add something more. This one does have the potential to do so but needs some reformatting and re-aligning.

6. The objective of this literature review needs to be far more clearly articulated. The objective should be clarified with regards to 'choice' ie from whose perspective? The patient? The prescriber? Based on outcomes? It is not completely clear. Whatever the objective is, the reader should not be surprised when they read e.g. the section on page 4 which describes how the results are separate into categories – currently, there is no clear connection.

7. I suggest that the authors consider reformatting the review snd perhaps reconsider the subheads e.g. rather than 'characteristic of available devices" use the heading "What is the difference between different devices". Also, instead of "Comparative clinical efficacy between device" replace with "Are some devices more effective in delivering medications to the lungs than others?" This is just a suggestion as I don't think the headings as they currently stand work.

8. Also difficult to tell which are major headings and which are subheading .e.g. it appears that Characteristics of available devices is a heading under which pMDIs, spacers and valved holding chambers etc and Comparative Clinical Efficacy between devices" all sit. I suggest a reformatting of the content. As an example perhaps something like the following:
   a. Characteristics of available devices (under which I would suggest including technical features and their impact on use).

b. Comparative clinical efficacy of different devices as drug delivery systems
c. Device Use in practice (to include common errors, device-specific errors, ‘critical’ errors technical features which impact on ability to use correctly, most common errors, patient-driven errors, which include ability to perform inhaler manoeuvres etc)
d. Recommendations for practice (to include recommendations for initial selection of devices, recommendations for adding on devices, recommendations for improving inhaler use over time)
e. Future developments, new technologies and research gaps.

Some more specific comments below as they relate to specific sections:

Methodology

9. Please include the keywords/search terms.

Results

10. The importance of inhaler device choice – I think this should be in the introduction, especially if a narrative around expert opinion beyond evidence is a significant part of this section. There are so many concepts raised in this section which don’t seem to add to the story but rather raise questions about what is important in device choice.

“The importance of Inhaler Choice”

11. I also think that the wording chosen needs to be more accurate to the data because in this field of research and practice, due to the difficulty in conducing high quality studies which answer specific questions about inhaler technique or ability to use, we have been far to ‘lose’ in the connections we draw from the available evidence to answer the questions we are really trying to answer. E.g. Page 5 “Patient acceptance and their ability of use certain devices is also important…..”. None of the references included in this statement ie references 14, 15 and 16 actually tested the patient’s ability to use. They all rely of patient-reported measures and they refer to adherence ie when the patient is likely to use but not their actual ability to use. Therefore, while this statement includes the words ‘may’ and ‘are likely’, it actually does not bring us any closer to the answer we are seeking.
12. The reporting of the Dephi work is over-emphasised and open to misinterprestaiton by the reader. Regarding the data relating to the Delphi consensus statements, once again this needs to be interpreted with caution. Traditionally Delphi surveys are administered to cohorts of experts and hence, it is regarded as an effective method for reaching consensus amongst EXPERTS and in this case, with regards to factors that impact on them selecting devices. Now the consensus has come up with something quite logical, that choice is driven by whether the patient can use the inhaler, previous experience, ease of teaching the inhaler etc etc However, when it comes using a device for the first time, how is the patient’s ability to use the device assessed? Is it trial and error? Is it what the prescriber perceives to be easy? And based on all that, can we then consider that some inhalers are actually easier to use? None of which were high recommendations of these Delphi panels???

13. Further to this, with regards to reference 16, with regards to ‘usability’ and ‘ease of training’, the particular reference is actually not able to determine this.
14. In addition to this, with regards to the experts views on costs, it is important to consider this with regards to the research of Laba et al., 2019 in JACI as it relates to the impact and importance of costs in relation to respiratory medication use by patients.
15. Therefore, I think this section on “The importance of inhaler choice” really should be about why choosing an inhaler is important and any actual evidence that proves this point – not what is currently included in this section. Alternatively, the heading for the section can be amended.

Characteristics of available devices

16. I think this should be in a table rather than text. If put in a table, I think it would be highly cited. This is not novel data but important references which are the basis of technology in this field.

Comparative clinical efficacy between devices

17. I would suggest that this section needs significant amending. This is a complex section, due to the challenges on actually getting data on the comparative efficacy of different devices therefore requires a more careful approach to presenting this to the reader. A more systematic approach to reporting the studies here needs to be taken. Either describe all the study designs or not. I actually think a table which includes study designs, comparison made and outcomes which summarise the results would be more helpful. The message is lost in all the text. There is a whole mix of comparisons, study designs, in vitro, clinical studies included here. A more systematic approach to reporting this is required.
18. In essence, there are actually only two comparisons that have been made, which can possibly give us insights into the difference in clinical efficacy related to device alone ie the comparison between the soft mist inhaler vs handihaler and the comparison between the Spiromax and the TH.
19. Please state in more detail why it is difficult to compare and summarise the difference between the clinical efficacy as it relates to devices alone. This is important for the non-expert reader. And also, how a real life retrospective database study can be used to give us insights. Ie why that specific design can be helpful here.
20. Please remove “Spiromax, which resembles a pMDI….”. It visually looks closer to an pMDI than anything else, but it should not be stated as being anything like a pMDI in an expert document like this. It does not technically resemble a pMDI and hence should not be stated as such in this manuscript. Perhaps one could say “For patients, the appearance of a Spiromax resembles the appearance of a pMDI hence in real-life studies, patients has tried to carry over some inhaler technique steps from the Spiromax to the pMDI, such as shaking the inhaler” I am certain the authors can selected better wording that I have suggested, but definitely need to reword the current text.
21. Finally, there is no conclusion to this section and I would suggest that the reader will need one.

Device handling and storage errors

22. I would suggest that the following subheadings in the following order would work better for the content:
    a. Incidence of inhaler technique errors,
    b. Common errors
    c. Device-specific errors
    d. Critical errors

Definition of critical errors

23. It should be made crystal clear that the only way to determine which errors are critical in real life is if it has been tested in a scientific way ie as per the Critikal study. Everything else, every other definition, consensus definition etc is just theory ! It does not mean anything until it is proven with scientific fact.

Common errors

24. It should be stated that the Sanchis interpretation of "poor inhaler technique" is a author-developed framework to enable the comparison of multiple studies with multiple devices in multiple patient populations over multiple time periods over the last 40 years.
25. "Numerous other studies and review have evaluated the effect of inhaler technique on asthma control." This sentence seems out of place – this section seems to be on what errors are common?? What does this sentence then refer to ? These studies should be included in the introduction relating to why inhaler technique is important not here?
26. There are many more and much better references to use than the Portuguese study noted here (41). Why was this study signalled out ?
27. I think that Table 2 should be the table of reference for this section should it not? Table 3 related to Device-specific errors, which appears in the next section? I think that Table 2 in Sanchis et al., 2016 or Table 1 in Bosnic-Anticevich et al., 2018, which is modified from Sanchis is more useful than the table provided. Are all these listed in Table 2 actually 'common' or 'possible' or 'reported' – this of course matters.

Device-specific errors

28. This section is difficult to follow. The reader just wants to know, which errors are specifically important for different inhalers. The authors include interventions and technique over time and effectiveness of different training is included – these are all distraction from what I think the reader will expect given the heading to this section. I think a good table with some explanation is all that is required here.
29. Further, there are so many studies that have published on the errors associated with different devices – what was the criteria for only selecting a handful here?
30. Specifically: "In a study of 180 COPD……correct use of pMDI…did not significantly differ.." What is meant by 'did not significantly differ..', with regards to what? The number of errors, the nature of the errors? This needs to be clarified.

Storage

31. If the studies related to FPD or DD then I think an explanation of these drug characteristics and humidity should be articulated up front.

32. “The difference in resistance to humidity..” why not just say “the impact of humidity on different DPIs…..” The term “resistance” and DPIs has connotations that do not relate to humidity.
33. “..single budesonide inhalers..” are you referring to budesonide-only containing DPIs??
34. These studies are not explained well.
35. What is the conclusion then, which devices are affected by storage conditions and which are not?

Shelf-life

36. What about the SMI? Does it not have an expiry date once opened?
37. Handihaler? Capsules?

Particle size considerations

38. Ok. So does particular size matter in real life and should this be a consideration when prescribing – what is the pragmatic approach here.
39. I think this section should be divided into theoretical impact of particle sizer and real-life studies on impact of particle size.

Inhalation flow rate

40. I like this section, however for the sake of being pragmatic, perhaps the recommendation by Seheult et al., that a spirometric PIFR of 196L/min should warrant further assessment of suitable of the device (at least the Diskus).
41. The explanation of reference 34 is not clear. It should also be noted that this study was in vitro. These are theoretical studies, when it comes to a pragmatic approach. This needs to be clear for the reader, because one could question the implications of invitro results for a pragmatic approach – perhaps important but not discriminatory to decision-making??? Not sure? But certainly has to be noted.

Inhalation volume

42. Why is inhalation volume described as a patient-related factor but inhalation flow rate/PIF is not?
43. What is the relevance of reporting all the regulatory issues. Are you saying, what are the implications for the reader and their decision to prescribe a particular inhaler?
44. I think it would be interesting for the authors to provide a hypothesis regarding why these differences are seen between the different devices and doses of medication?

The Need for patient education

45. The heading, ”The need for patient education” does not fit the content that follows. The need for patient education should be a fundamental recommendation. Perhaps this section should have the following heading: “Patient knowledge, perceptions and behaviours” . I need to think more carefully about how the multi inhalers section fits into this? Maybe should be a separate subheading of its own?

Patient perceptions and behaviours

46. Concluding that Levy et al., 2016, or in fact the primary reference by Chorao et al., 2014 concludes that patient preference makes a difference to outcomes is just not

correct. This needs to be amended. Chorao et al., 2014 comes to the conclusion, as does Levy et al., 2016, that there is no conclusive evidence that patient satisfaction impacts on outcomes. The current text may mislead the reader – it needs to be amended.

47. Are the finding report from Norderud et al., 2016 related to behaviour or would we put this down to knowledge?? Heading should therefore include 'knowledge'.
48. "Factors associate with poor inhaler technique…." Would have been a nice statement to start this section with, rather than para 2?? What does 'serious' error mean when we know that the only error that can be considered critical is that proven by evidence (Price et al and Critikal).

Device-specific errors

49. Do we need to have this heading again? Is there no more appropriate heading for this section? It seems to be more about education and lack of knowledge of certain steps – would that not be a better heading?

New technologies

50. This is now focusing on adherence and to this point, while adherence has been noted by the authors as it relates a handful of manuscripts, it does not fit here – it needs an explanation and context.
51. Firstly there needs to be some comment about what the purpose is of new technologies, what problems are they trying to address and why trying to solve these problems by placing technology in inhalers is important and relevant. Then the technologies associated with the various problems need to be addressed, systematically.

Interventions to improve inhaler technique

52. This does not fit here??

53. Finally, I would like to see a series of recommendations which the authors feel are hierarchical in terms of selecting devices.
