# Supplementary material for: What to consider before prescribing inhaled medications: a pragmatic approach for evaluating the current inhaler landscape
Source: Ther Adv Respir Dis. 2019 Dec 6;13:1753466619884532. doi: 10.1177/1753466619884532 (PMC6900625; doi:10.1177/1753466619884532)
Supplement: Supplemental_material – Supplemental material for What to consider before prescribing inhaled medications: a pragmatic approach for evaluating the current inhaler landscape [file Supplemental_material.pdf]

Appendix

Table S1: Search terms used

Search term	Individual terms used
**Indication**	Asthma* or (COPD or “chronic obstructive pulmonary disease”)
**At least one device type or brand name**	inhaler* or inhalat* or device* or aerosol* or “inhal* device*” or “dry-powder inhaler*” or DPI* or “pressurised metered dose inhaler*” or pMDI* or “metered-dose inhaler*” or “metered dose inhaler*” or MDI* or “hydrofluoroalkane inhaler*” or HFA* or “CFC-free inhaler*” or “CFC free inhaler*” or “soft mist inhaler*” or “SMI” or spacer or “holding chamber” or “valved holding chamber” or “intelligent inhaler*” or “smart inhaler*” or “intelligent device*” or “smart device*” or diskus or accuhaler or handihaler or ellipta or aerolizer or cyclohaler or diskhaler or rotadisk or clickhaler or easyhaler or neohaler or pressair or MAGhaler or novolizer or pulvinal or spinhaler or spiromax or turbohaler or turbuhaler or twisthaler or NEXThaler or genuair or breezhaler or forspiro or rotahaler or aerosphere or modulate or evohaler or autohaler or easi-breathe or respiclick or respimat
**At least one keyword or phrase**	“inhaler handling” or “device handling” or “handling error*” or “critical handling error*” or “critical error*” or “inhal* technique*” or “inhal* error*” or “device error*” or “expir* date*” or shelf-life or shelf life or “drug deliv*” or “deliv* dos*” or “fine-particle dos*” or extrafine or extra-fine or “fine particle fraction*” or “particle size*” or “mass median aerodynamic diameter” or “inhal* flow rate*” or “inhal* flowrate*” or “air-flow rate*” or “airflow rate*” or “flow rate*” or “inspirit* flow” or “inhal* volume*” or “lung delivery” or “lung deposition” or (deposition and lung*) or ((“drug stor*” or storage or store* or storing) and (moisture or humid*))
